# Comparing Effectiveness of HRV-Biofeedback and Mindfulness for Workplace Stress Reduction: A Randomized Controlled Trial

**DOI:** 10.1007/s10484-020-09477-w

**Published:** 2020-06-18

**Authors:** Amelie Edith Brinkmann, Sophia Antonia Press, Eduard Helmert, Martin Hautzinger, Inna Khazan, Jan Vagedes

**Affiliations:** 1 Department of Clinical Psychology and Psychotherapy, Institute of Psychology, University of Tuebingen, 72070 Tübingen, Germany; 2 ARCIM Institute for Academic Research in Complementary and Integrative Medicine, 70794 Filderstadt, Germany; 3 Harvard Medical School, Boston, MA USA; 4 Department of Neonatology, University Hospital Tuebingen, 72070 Tübingen, Germany

**Keywords:** Stress, Occupational health, Heart rate variability biofeedback, Mindfulness meditation

## Abstract

Psychophysiological disorders due to work-related stress continue to be highly costly for health systems and approaches for cost-effective and easily accessible interventions are much needed. Both heart rate variability-biofeedback (HRV-Bfb) and mindfulness-based interventions (MBI) have been empirically shown to reduce stress. This study compares these two interventions in the work context to a wait-list-control-group (WLC). In this three-armed randomized controlled trial (RCT), 69 healthy adults employed in the same organization were randomized to participate in HRV-Bfb, MBI or the WLC. Participants were assessed for psychophysiological parameters of stress (stress perception, coping, HRV parameters and cortisol) and stress related symptoms (depressive symptoms, psychological wellbeing, mindfulness and self-compassion). Participants trained using either HRV-Bfb or MBI for 6 weeks on a daily basis. Outcomes were assessed at baseline, after the intervention and at follow-up 12 weeks later. Results did not show any statistically significant differences between HRV-Bfb and MBI groups, and neither of the intervention groups (IGs) differed from the WLC. Findings suggest an overall reduction in stress for all groups, including the WLC, with mostly small to medium effect sizes. However, it is important to note that participants with higher baseline stress levels might benefit more from mindfulness and biofeedback-based stress reduction interventions. The results have to be interpreted with caution due to the relatively small sample size. MBI might have a slightly stronger effect on stress reduction in comparison to HRV-Bfb, as suggested by the effect sizes. This study highlights issues and challenges of the implementation of such interventions in corporate health management.

## Introduction

Work-related stress contributes to various psychophysiological disorders such as cardiovascular disease, neck pain, shoulder pain, and symptoms of anxiety and depression (Fishta and Backé 2015; Eurofound 2016; Marcatto et al. 2016). Several biomarkers have been shown to be reliable indicators of increased stress. For example, cortisol is a steroid hormone released during the stress response. Heart rate variability (HRV) is the variation in time between consecutive heart beats (RR-intervals) and serves as quantitative marker of autonomic balance and physiological stress (Task Force of The European Society of Cardiology and the North American Society of Pacing and Electrophysiology 1996). Higher HRV indicates greater parasympathetic activity and better self-regulation, and is associated with lower cardiovascular risk (Kiviniemi et al. 2010) and improved cognitive performance (Moore et al. 2011). Lower HRV is related to difficulty in self-regulation and poorer cardiovascular health. Several studies have shown that job stress reduces HRV (Vrijkotte et al. 2000; Chandola et al. 2010).

Stress-related disorders continue to be highly costly to economies and health systems by causing up to half of all work absences (absenteeism) and loss of productivity due to working while sick (presenteeism; European Agency for Safety Health at Work 2013). Therefore, new approaches for cost-effective as well as accessible interventions in stress-reduction in the workplace are much needed. A technique that may be used in a self-directed way independent of time and location in the workplace is especially important as employees often indicate organizational problems such as a lack of time or support factors for not attending psychological interventions provided in organizations (Dreison et al. 2015; Bartlett et al. 2017). Two interventions that meet these criteria and could provide help for large groups of people are heart rate variability biofeedback training (HRV-Bfb) and mindfulness-based interventions (MBI).

HRV-Bfb is a well-established, empirically supported technique for improving self-regulation and alleviating symptoms of stress, anxiety, and other psychophysiological disorders (Prinsloo et al. 2013; Sutarto et al. 2012; Wells et al. 2012; Lewis et al. 2015). During HRV-Bfb training individuals learn to breathe at the optimal respiratory frequency to maximally increase their HRV (Moore et al. 2011; Prinsloo et al. 2013). A recent meta-analysis from Goessl et al. (2017) affirms the efficacy of HRV-Bfb with wearable devices on self-reported stress. However, only eight studies examining stress symptoms as an outcome parameter for healthy individuals with only three in a workplace context could be included, reflecting a lack of research in this field. Also, Goessl et al. (2017) suggest the need to evaluate HRV-Bfb interventions with a format other than self-reported measures and to include follow-up measures to decrease reporting bias and examine long-term effects. De Witte et al. (2019) emphasize the need for more studies including both psychological and physiological parameters of stress in their review on the effectiveness of general biofeedback trainings on psychophysiological outcomes of stress. They also included four RCTs using HRV biofeedback in the work context with healthy adults. The review describes preliminary evidence of the effectiveness of the trainings despite a large diversity in intervention design and effectiveness. MBIs also improve the regulation of the autonomic and central nervous systems as indicated by HRV (Azam et al. 2016; Krygier et al. 2013). Jon Kabat-Zinn (1990) developed the first MBI for stress reduction, a program called Mindfulness-Based Stress Reduction (MBSR). MBSR emphasizes non-judgmental attitude and includes formal and informal meditation practices as well as hatha yoga. Today, there exists several different forms of MBIs at the workplace, mostly varying in their conceptualization of mindfulness and the duration of intervention (Jamieson and Tuckey 2016; Klatt et al. 2016). Recent reviews from Janssen et al. (2018), Jamieson and Tuckey (2016) and Sharma and Rush (2014) show positive effects of MBIs on self-reported stress in the workplace for healthy adults. However, these authors also note a lack of psychophysiological measures, randomized-controlled trials (RCTs), and evaluation of long-term effects of these interventions. Both HRV-Bfb and MBIs (Sanada et al. 2016; O’Leary et al. 2015; Bouchard et al. 2012) have been shown to decrease cortisol. Workplace interventions such as HRV-Bfb and MBIs can also improve psychological indicators of stress such as perceived stress, coping with stress, depression or anxiety (Burton et al. 2016; Dobie et al. 2015; dos Santos et al. 2016; Henriques et al. 2011; Hülsheger et al. 2013; Jamieson and Tuckey 2016; Klatt et al. 2009; Koncz et al. 2016; Lemaire et al. 2011; Ratanasiripong et al. 2012; Ratanasiripong et al. 2015; Schroeder et al. 2016; Sutarto et al. 2012; Taylor et al. 2015; Van der Zwan et al. 2015; Wells et al. 2012).

Few studies have examined the difference between the two methods of stress reduction, both of which emphasize the focusing of attention and calm breathing exercises. Van der Zwan et al. (2015) compared the effectiveness of both HRV-Bfb and MBI on stress reduction in their study with stressed healthy adults but didn’t find any significant differences between the interventions. They reported an overall positive effect of both interventions on subjective stress reduction. This study however did not include a control group, making it difficult to make conclusions regarding the effectiveness of the two methods in reducing stress. Ratanasiripong et al. (2015), who also compared the effectiveness of the two interventions, included a control group and measured perceived stress and anxiety in nursing students. They found a reduction of perceived stress only for MBI, not for HRV-Bfb. However, this difference was not significant.

To our knowledge, there aren’t any studies which compare HRV-Bfb and MBI in a workplace setting of corporate health management using both psychological and psychophysiological assessments. Therefore, the aim of this study is to examine the effect of both interventions on psychological and psychophysiological measures of stress compared to a waiting-list control group (WLC) in a workplace setting. We predicted that both intervention groups will experience a reduction in stress levels compared to the WLC group.

## Methods

### Study Design

In this three-armed RCT with repeated measures, healthy adults employed in the same organization were randomized to participate in HRV-Bfb, MBI or the WLC. The WLC later received a combined training (mindfulness-based biofeedback, MBB, not taken further account in the present work). The Ethics Committee of the University of Tuebingen in Germany approved of the study (682/2014BO2), which was preregistered at ClinicalTrials (NCT02709551). All participants gave informed consent.

### Participants and Procedure

Recruiting took place in the Staatstheater Stuttgart on several occasions, such as health day and assemblies of the theatre. We also sent an e-mail with information concerning the study to all employees (e.g. orchestra, choir and administration). Those individuals who gave us their personal information, were contacted via telephone to complete a short screening to make sure that participation on the required date was possible. Participants were also asked to complete an online 12-item screening questionnaire of the German questionnaire*Trierer Inventar zum chronischen Stress* (TICS: Trier Inventory for Chronic Stress; Schulz et al. 2006) to assess their general level of perceived stress. All participants matching the time inclusion criterion were invited to an in vivo clinical interview screening with the *Mini-DIPS* (Margraf 2013) to assess psychopathology. Participants were randomized to one of the three conditions at that time. Participants were stratified by gender and perceived stress prior to randomization to ensure there was an even distribution of gender and perceived levels of stress among participants in all three groups. Randomization was carried out using opaque envelopes (1 = HRV-Bfb, 2 = MBI or 3 = WLC).

Sample size was calculated a priori using G*Power (Faul et al. 2007) with α = 0.05, β = 0.95 and three points of measurement for a within–between repeated measures analysis of variance (ANOVA) with an expected effect size of *f* = 0.25. A total sample size of N = 54 was calculated.

Data collection took place at the workplace in a separate room. It was conducted by trained personnel. The trainings for the respective intervention method took place over four consecutive half days. Participants practiced their skills independently after the initial training for a period of 6 weeks. The follow-up measurements were assessed after 12 weeks. Data collection on the assessment points took about an hour.

### Interventions

The trainings were guided by experienced trainers of our team in either HRV-Bfb or MBI. Participants were instructed to practice half an hour daily and to complete a self-report each day to monitor their configurations (HRV-Bfb), kind of exercises (MBI) and for how long they had practiced. Participants were contacted twice (after weeks 1 and 3) to monitor their progress. Booster sessions for the clarification of general or technological questions were offered two times during the six weeks of training (after weeks 1 and 3).

### Heart Rate Variability-Biofeedback

HRV-Bfb training consisted of a psychoeducation about the physiology of stress and the relationship between stress and heart rate variability, as well as instruction in the use of the mobile HRV training device called “Qiu” (BioSign GmbH, Ottenhofen, Germany). The Qiu is a spherical hand-held battery-operated device. The palm and fingers cover the lower half of the sphere so that the pulse rate can be measured at the palm or one digit of the hand by optical pulse sensors. HRV is automatically calculated from the pulse rate. The upper half of the Qiu provides feedback via a spectrum of red (low HRV) to green (high HRV) light. Individually adjustable blue LED light signals indicate the breathing frequency helping the individual to determine their resonance frequency breathing rate which corresponds to maximal HRV. Resonance frequency breathing rate is typically in the range of 4.5 to 7 breaths/min, with an average of 6 (Lehrer et al. 2000). Participants also could use an ear clip to measure pulse if wished so. The Qiu stores heart rate measures along with the time and duration of each practice sequence. It can be connected via USB port to a computer to transmit data, using specially developed software (‘HRV-Scanner’; BioSign GmbH, Ottenhofen, Germany). HRV-Bfb exercises consisted of slow breathing either following the pacer or independently and experimenting in changing breathing to maximize HRV (using the feedback provided by the light).

### Mindfulness-Based Intervention

The MBI was based on Mindfulness-Based Stress Reduction (MBSR) by Kabat-Zinn (1990) but also included elements of self-compassion, acceptance and commitment therapy and Mindfulness-Based Cognitive Therapy (MBCT; Khazan 2013) and consisted of formal guided meditations and informal exercises. Examples of formal guided meditations include mindfulness of the breath and mindfulness of thoughts, feelings, and physiological sensations. Informal meditation practices encouraged brief pauses throughout the day during which participants would volitionally shift their attention to present moment awareness without judging. Participants were given meditation CDs consisting of 12 guided meditations which were recorded by a member of our team to support formal meditation at home after the training provided by the study.

### Outcomes

Primary outcomes were psychological and physiological parameters of stress: stress perception, coping, HRV parameters and cortisol. Secondary outcomes were depressive symptoms, psychological wellbeing, mindfulness and self-compassion. Psychological outcomes were assessed via online surveys. Data collection took place from February to July 2015 in Stuttgart, Germany.

### Psychological Assessment

To assess the primary psychological outcomes, two questionnaires were used to record parameters of stress: the scale *chronic stress* of the TICS was used to assess perceived stress as a global parameter, whereas the German questionnaire *Stressverarbeitungsfragebogen* (SVF-120: stress-coping questionnaire; Janke and Erdmann 1997) assessed stress coping. Two scales for positive (*POS*) and negative (*NEG*) coping strategies were included. Secondary psychological parameters were measured using the German version of the *Beck-depression inventory* (BDI-II; Hautzinger et al. 2006) for depressive symptoms, two modules of the *Hamburg Modules for the Assessment of Psychosocial Health in Clinical Practice* (HEALTH-49; Rabung et al. 2009) to record psychological wellbeing (psychological and somatoform complaints and restrictions on activity and participation); the short version of the *Freiburg Mindfulness Inventory *(FFA-14; Walach et al. 2006) to record mindfulness; and the short German version of the of the *self-compassion scale* (SCS; Hupfeld and Ruffieux 2011) to assess self-compassion. Internal consistency at pretest was sufficient to very good (chronic stress α = 0.84; positive coping strategies 0.66 ≤ α ≤ 0.88 and negative coping strategies 0.81 ≤ α ≤ 0.91; depressive symptoms α = 0.89; psychological and psychosomatic complaints α = 0.83 and participation and activation α = 0.80; mindfulness α = 0.80 and self-compassion = 0.76).

### Measures of Heart Rate Variability

#### Recording and Analysis

Participants were asked to abstain from drinking beverages with caffeine or alcohol as well as smoking the day of their assessment. Two electrodes and an ear clip were applied to measure ECG and pulse wave. After a short resting phase, measurements started with a deep-breathing test (parasympathetic functional testing), a 1-min respiratory sinus arrhythmia (RSA) measurement during which participants were asked to breathe following a visual pacer set to 6 breaths/min This was followed by a 5-min short-term HRV-test (observation of the resting regulation) during which participants were asked to breathe calmly at their own pace. All HRV data were sampled, filtered and analyzed with the HRV-Scanner software (BioSign GmbH, Ottenhofen, Germany).

#### HRV-Parameters

Two methods of measuring HRV are used as representative parameters of HRV: root mean square of the squared differences of the RR intervals of successive heartbeats (RMSSD) and the standard deviation of the RR intervals (SDNN). SDNN is a general measure of HRV, while RMSSD provides an indication of parasympathetic activity in particular.

### Cortisol

Participants independently took their saliva sample. To be consistent with prior research, we focused on morning cortisol levels. Participants were asked to take the sample directly after waking up and to record the exact time of retrieval. Saliva samples were then collected at the workplace and sent to a laboratory for analysis using standard procedures [liquid chromatography–mass spectrometry (LC–MS)-method].

### Statistical Methods

Differences between groups at pre-test were analyzed using Pearson’s Chi square Test (categorical data) or a one-way ANOVA. If the cell occupation was too low, Fisher’s Exact Test was used. Normality of distribution of the data was tested using the Kolmogorov–Smirnov Test. In case of violation of the assumption of normal distribution non-parametric tests were used to check for differences. There were no missing data. The effect of time and differences between groups were assessed using 3 (assessment points) × 3 (groups) repeated measures ANOVAs. Within-group-analysis were conducted using repeated measures ANOVAs for the factor “time”. Sphericity was tested using Mauchly’s Test. In case of violation of sphericity, Greenhouse–Geisser corrected values were considered. Pairwise comparisons were conducted using Bonferroni-corrected post hoc tests. Two-sided *p*-values of < 0.05 were considered statistically significant. Effect sizes for ANOVAs were calculated using partial eta-squared (*η*²) and Cohen’s *d* was used for comparison of effect sizes. Effect sizes *d* = 0.2–0.4 were considered small, 0.5–0.8 medium and > 0.8 large. Effect sizes smaller *d* = 0.2 were considered as no effect. *η*² < 0.06 was considered small, 0.06–0.14 medium and > 0.14 large. All analyses were conducted using SPSS version 24.0.

## Results

### Study Sample

Recruiting took place in the Opera House and Theatre of Stuttgart from November 2014 to March 2015. The time frame of the study ranges from November 2014 to July 2015 for data collection.

**Fig. 1:**
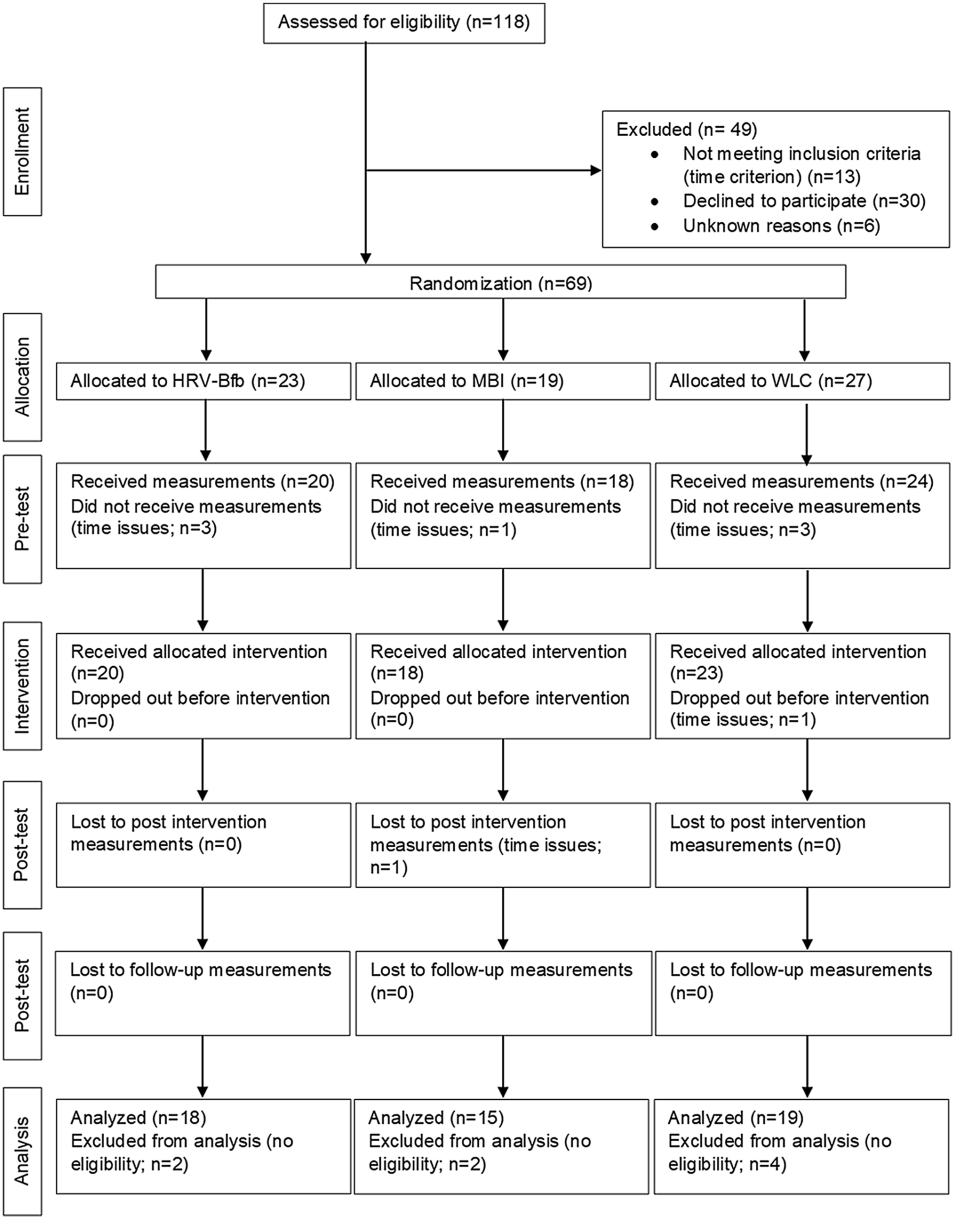
CONSORT diagram of participant flow through the study. *HRV-Bfb* heart rate variability-biofeedback, *MBI* mindfulness-based intervention, *WLC* wait-list-control-group

In total, N = 118 employees were screened for eligibility (see Fig. 1). Sixty-nine employees were included in the study of whom 61 received an intervention. Data from 52 participants were analyzed. Excluded from the analysis were those participants who reported psychological illness (n = 5) or physical illness/heart rate or cortisol altering medications (n = 3). One participant was excluded because he dropped out of the study after the intervention (n = 1). A diagram of participant flow is presented in Fig. 1.

There were no statistically significant differences in baseline characteristics between the intervention groups and the WLC, neither in demographic nor in primary or secondary outcome parameters (Tables 1, 2). 37 (71.2%) of the participants were female. Mean age of all participants was 43.27 (*SD* = 10.45) years. At baseline, 28 (53.8%) participants had experience in meditation but only 8 (15.4%) of them had experience in mindfulness meditation. Only 5 (9.6%) practiced some form of meditation frequently, while no 1 practicing mindfulness on a regular basis. Participants worked in different professional fields within the theater, including orchestra, choir, creative employees, staff council/social services department, venue staff, technology, merchandising, and administration. 39 (75%) of the participants exercised at least once a week, 9 (17.3%) were regular smokers and 13 (25%) drank alcohol on a regular basis. During the intervention phase, participants practiced their respective training method on average 18.09 (*SD* = 7.13) min daily. Over the 6 training weeks, the HRV-Bfb group practiced on average 19.74 (*SD* = 7.01) min/day and the MBI group practiced on average 16.11 (*SD* = 6.97) min/day. Mean practice duration did not significantly differ between the groups [*F *(1, 31) = 2.2, *p* = .148, *η*² = 0.066]. To exclude possible social desirability effects in self-report for the practice time, we compared the reported HRV-Bfb exercise time with the actual exercise time stored in the Qiu. Data matched, which means participants reported their exercise time accurately. There were no such treatment fidelity measures available for MBI, but we did not expect there to be any differences between the groups in truthful reporting practice time.

**Table 1:** Percentages (frequencies) and test statistics of the demographic characteristics for participants randomized to HRV-Bfb, MBI and the WLC

Variable	HRV-Bfb (n = 18)	MBI (n = 15)	WLC (n = 19)	Test statistics (*df*)	*p*
Sex				*χ*^2^(2) = 0.34	0.863
Female	66.7 (12)	73.3 (11)	73.7 (14)		
Male	33.3 (6)	26.7 (4)	26.3 (5)		
Age	42.06 (*SD* = 11.96)	45.20 (*SD* = 8.4)	42.89 (*SD* = 10.71)	*F*(2) = 0.38	0.686
Previous experience meditation				*χ*^2^(2) = 1.42	0.536
Experience	50.0 (9)	66.7 (10)	47.4 (9)		
No experience	50.0 (9)	33.3 (5)	52.6 (10)		
Mindfulness meditation				*χ*^2^(2) = 1.58	0.577
Mindfulness	11.1 (2)	13.3 (2)	21.1 (4)		
Other	38.9 (7)	53.3 (8)	26.3 (5)		
Meditation on regular basis				*χ*^2^(2) = 1.43	0.571
Regularly	5.6 (1)	20 (3)	5.3 (1)		
Not regularly	44.4 (8)	46.7 (7)	42.1 (8)		
Sports				*χ*^2^(4) = 1.4	0.882
No sports	16.7 (3)	33.3 (5)	26.3 (5)		
1 ×/week	38.9 (7)	33.3 (5)	36.8 (7)		
2–5 ×/week	44.4 (8)	33.3 (5)	36.8 (7)		
Smoking				*χ*^2^(2) = 0.56	0.812
Yes	22.2 (4)	13.2 (2)	15.8 (3)		
No (not currently)	77.8 (14)	86.7 (13)	84.2 (16)		
Alcohol consumption				*χ*^2^(2) = 3.28	0.204
Yes	11.1 (2)	26.7 (4)	36.8 (7)		
No (not currently)	88.9 (16)	73.3 (11)	63.2 (12)		
Marital status				*χ*^2^(4) = 1.84	0.910
Solo	36.9 (7)	46.7 (7)	21.1 (4)		
Married/in a relationship	27.8 (5)	6.7 (1)	15.8 (3)		
Separated	33.3 (6)	46.7 (7)	63.2 (12)		
Children				*χ*^2^(2) = 0.75	0.733
Children	38.9 (7)	46.7 (7)	52.6 (10)		
No children	61.1 (11)	53.3 (8)	47.4 (9)		
Level of education				*χ*^2^(4) = 5.41	0.241
Highschool diploma	27.8 (5)	6.7 (1)	15.8 (3)		
No highschool diploma	38.9 (7)	46.7 (7)	21.1 (4)		
University	33.3 (6)	46.7 (7)	63.2 (12)		

*HRV-Bfb* heart rate variability biofeedback, *MBI* mindfulness-based intervention, *WLC* waiting-list control, *SD* standard deviation

**Table 2:** Overall time and group effects and Cohen’s *d* (CI*d*) within-group effect sizes for stress and stress-related symptoms

Variable	Group	Overall time effect							Interaction time × group		
		*F *(*df*_*M*_*,* *df*_*R*_)	*p*	η²	T0–1		T0–2		*F *(*df*_*M*_, *df*_*R*_)	*p*	η²
					*d*	CI*d* (95%)	*d*	CI*d* (95%)			
TICS-SSCS	HRV-Bfb	9.34 (2, 98)	< 0.001	0.16	− 0.44	[− 1.1, 0.22]	− 0.28	[− 0.93, 0.38]	0.57 (4, 98)	0.687	0.02
	MBI				− 0.48	[− 1.21, 0.24]	− 0.26	[− 0.98, 0.46]			
	WLC				− 0.37	[− 1, 0.28]	0.02	[− 0.62, 0.66]			
SVF 120											
POS	HRV-Bfb	2.81 (2, 98)	0.065	0.05	0.01	[− 0.65, 0.66]	0.25	[− 0.41, 0.9]	0.25 (4, 98)	0.908	0.01
	MBI				− 0.06	[− 0.78, 0.66]	0.06	[− 0.66, 0.77]			
	WLC				− 0.14	[− 0.77, 0.5]	0.13	[− 0.51, 0.77]			
NEG	HRV-Bfb	1.58 (2, 98)	0.211	0.03	0.02	[− 0.63, 0.67]	− 0.05	[− 0.7, 0.61]	2.83 (4, 98)	0.029	0.1
	MBI				− 0.33	[− 1.05, 0.39]	− 0.24	[− 0.96, 0.48]			
	WLC				0.09	[− 0.55, 0.72]	0.05	[− 0.59, 0.68]			
HRV											
RMSSD											
RSA	HRV-Bfb	2.82 (2, 98)	0.064	0.05	0.09	[− 0.56, 0.74]	− 0.07	[− 0.73, 0.58]	0.3 (4, 98)	0.88	0.01
	MBI				0.25	[− 0.47, 0.97]	− 0.24	[− 0.96, 0.48]			
	WLC				0.26	[− 0.38, 0.9]	− 0.04	[− 0.67, 0.6]			
Short-term	HRV-Bfb	1.19 (1.48, 72.71)	0.3	0.02	0.12	[− 0.54, 0.77]	0.14	[− 0.52, 0.79]	0.28 (3, 72.7)	0.836	0.01
	MBI				0.2	[− 0.52, 0.92]	0.21	[− 0.51, 0.92]			
	WLC				0.25	[− 0.39, 0.89]	0.16	[− 0.48, 0.8]			
SDNN											
RSA	HRV-Bfb	3.26 (2, 98)	0.043	0.06	0.06	[− 0.59, 0.71]	0.07	[− 0.58, 0.73]	0.74 (4, 98)	0.566	0.03
	MBI				0.38	[− 0.35, 1.1]	− 0.15	[− 0.86, 0.57]			
	WLC				0.31	[− 0.33, 0.95]	0.05	[− 0.59, 0.68]			
Short-term	HRV-Bfb	5.05 (1.55, 75.98)	0.015	0.09	0.4	[− 0.26, 1.06]	0.52	[− 0.14, 1.19]	1.14 (3.1, 75.98)	0.339	0.04
	MBI				− 0.06	[− 0.77, 0.66]	0.1	[− 0.61, 0.82]			
	WLC				0.39	[− 0.25, 1.03]	0.48	[− 0.16, − 1.13]			
Cortisol	HRV-Bfb	12.78 (2, 98)	< 0.001	0.21	− 0.82	[− 1.5, − 0.14]	− 0.05	[− 0.71, 0.6]	0.39 (4,98)	0.817	0.02
	MBI				− 1.56	[− 2.37, − 0.74]	− 0.37	[− 1.09, 0.35]			
	WLC				− 0.72	[− 1.38, − 0.07]	0.15	[− 0.49, 0.79]			
BDI-II	HRV-Bfb	10.49 (1.54, 75.39)	< 0.001	0.18	− 0.62	[− 1.29, 0.05]	− 0.05	[− 1.11, 0.21]	0.55 (3.08, 75.39)	0.654	0.02
	MBI				− 0.88	[− 1.62, − 0.13]	− 0.86	[− 1.61, − 0.11]			
	WLC				− 0.34	[− 0.98, 0.3]	− 0.7	[− 1.36, − 0.05]			
HEALTH-49 A	HRV-Bfb	8.42 (2, 98)	< 0.001	0.15	− 0.4	[− 1.06, 0.26]	− 0.33	[− 0.98, 0.33]	0.61 (4, 98)	0.658	0.02
	MBI				− 0.32	[− 1.04, 0.4]	− 0.44	[− 1.17, 0.28]			
	WLC				− 0.18	[− 0.82, 0.46]	− 0.42	[− 1.06, 0.22]			
HEALTH-49 E	HRV-Bfb	3.28 (2, 98)	0.042	0.06	− 0.2	[− 0.86, 0.45]	− 0.06	[− 0.72, 0.59]	0.82 (4, 98)	0.513	0.03
	MBI				− 0.56	[− 1.29, 0.17]	− 0.46	[− 1.18, 0.27]			
	WLC				− 0.13	[− 0.76, 0.51]	− 0.49	[− 1.14, 0.15]			
FFA-14	HRV-Bfb	8 (2, 98)	0.001	0.14	0.24	[− 0.41, 0.9]	0.19	[− 0.46, 0.85]	3.27 (4, 98)	0.015	0.12
	MBI				0.62	[− 0.11, 1.36]	0.86	[0.11, 1.6]			
	WLC				− 0.18	[− 0.81, 0.46]	0.2	[− 0.44, 0.83]			
SCS-D	HRV-Bfb	6.05 (1.77, 86.9)	0.005	0.11	0.05	[− 0.6, 0.71]	0.24	[− 0.41, 0.9]	1.88 (3.55, 86.9)	0.129	0.07
	MBI				0.54	[− 0.19, 1.26]	0.57	[− 0.16, 1.31]			
	WLC				− 0.03	[− 0.67, 0.61]	0.1	[− 0.53, 0.74]			

*TICS-SSCS* scale *chronic stress* of the Trier Inventory for Chronic Stress, *SVF 120 POS* scale *positive coping strategies* of the stress-coping questionnaire, *SVF 120 NEG* scale *negative coping strategies* of the stress-coping questionnaire, *HRV* heart rate variability, *RSA* respiratory sinus arrhythmia, *RMSSD* root mean square of the squared differences of the RR intervals of successive heartbeats, *SDNN* standard deviation of the RR intervals, *BDI-II* Beck-depression inventory, *HEALTH-49 A and HEALTH-49 E* module A (psychological and somatoform complaints) and module E (restrictions on activity and participation) of the Hamburg Modules for the Assessment of Psychosocial Health in Clinical Practice, *FFA 14* short version of the Freiburg Mindfulness Inventory, *SCS-D* short German version of the of the self-compassion scale, *HRV-Bfb* heart rate variability-biofeedback (n = 18), *MBI* mindfulness-based intervention (n = 15), *WLC* wait-list-control-group (n = 19)

### Intervention Effects

Observed means (*SD*) for all points of measurement are presented in Table 3, test statistics and effect sizes in Table 2. Primary outcome parameters are chronic stress as a global parameter for stress perception (TICS), positive and negative coping strategies (SVF-120; Coping POS/NEG), RMSSD and SDNN (RSA and short-term) for HRV as well as cortisol. Secondary outcome parameters include depressive symptoms (BDI-II), psychological wellbeing [module A (psychological and somatoform complaints) and E (restrictions on activity and participation) of the HEALTH-49], mindfulness (FFA-14) and self-compassion (SCS-D).

**Table 3:** Observed means (*SD*) for stress and stress related symptoms

Measure	Group	T0		T1		T2	
		*M*	*SD*	*M*	*SD*	*M*	*SD*
TICS-SSCS							
	HRV-Bfb	17.22	8.29	13.83	7.08	15	7.67
	MBI	17.67	6.6	14.53	6.36	16.07	5.73
	WLC	20.47	7.89	17.74	7.13	20.63	8.33
SVF 120							
POS	HRV-Bfb	11.98	2.12	12	2.43	12.52	2.29
	MBI	11.69	2.7	11.52	2.87	11.85	2.96
	WLC	11.41	2.65	11.03	2.94	11.74	2.41
NEG	HRV-Bfb	9.8	2.8	9.86	2.89	9.67	2.82
	MBI	10.22	3.45	9.06	3.55	9.36	3.68
	WLC	10.86	4.17	11.23	4.32	11.04	3.52
HRV							
RMSSD							
RSA	HRV-Bfb	38.62	22.15	40.88	27.97	37.2	15.91
	MBI	37.12	15.44	41.24	17.55	33.51	14.91
	WLC	46.86	24.78	55.79	41.63	45.98	25.87
Short-term	HRV-Bfb	29.66	18.02	31.7	17.24	31.84	13.93
	MBI	28.95	12.26	31.16	9.39	31.37	11.19
	WLC	36.47	20.88	44.28	44.94	39.67	26.91
SDNN							
RSA	HRV-Bfb	65.3	31.15	67.36	37.17	75.51	23.82
	MBI	63.56	22.7	73.02	27.46	88.17	23.92
	WLC	75.51	34.25	60.16	47.04	77.01	31.49
Short-term	HRV-Bfb	50.14	26.7	62.99	26.45	67.37	24.22
	MBI	57.61	32.36	56.88	18.72	62.62	52.69
	WLC	59.29	30.31	72.3	21.16	71.1	33.37
Cortisol	HRV-Bfb	2.63	1.44	1.5	1.33	2.55	1.64
	MBI	2.72	1.08	1.28	0.74	2.21	1.62
	WLC	2.41	1.71	1.41	0.95	2.69	2.05
BDI-II	HRV-Bfb	8.67	6.66	4.67	6.31	5.33	8.13
	MBI	7.33	4.46	3.67	3.89	3.2	5.11
	WLC	7.95	5.96	6.26	3.82	4.26	4.26
HEALTH-49 A	HRV-Bfb	0.65	0.42	0.49	0.37	0.52	0.38
	MBI	0.58	0.32	0.47	0.37	0.44	0.31
	WLC	0.78	0.57	0.67	0.64	0.56	0.47
HEALTH-49 E	HRV-Bfb	1.36	0.89	1.19	0.79	1.32	0.69
	MBI	1.42	0.52	1.08	0.68	1.13	0.73
	WLC	1.33	0.65	1.24	0.76	1.05	0.47
FFA-14	HRV-Bfb	37.5	4.65	38.67	5.98	38.44	5.64
	MBI	35.2	4.99	37.93	3.67	39.53	5.97
	WLC	37.4	5.08	36.42	5.14	38.58	6.8
SCS-D	HRV-Bfb	3.25	0.44	3.27	0.5	3.36	0.67
	MBI	3.26	0.33	3.5	0.39	3.52	0.67
	WLC	3	0.46	2.98	0.4	3.07	0.69

*TICS-SSCS* scale *chronic stress* of the Trier Inventory for Chronic Stress, *SVF 120 POS* scale *positive coping strategies* of the stress-coping questionnaire, *SVF 120 NEG* scale *negative coping strategies* of the stress-coping questionnaire, *HRV* heart rate variability, *RSA* respiratory sinus arrhythmia, *RMSSD* root mean square of the squared differences of the RR intervals of successive heartbeats, *SDNN* standard deviation of the RR intervals, *BDI-II* Beck-depression inventory, *HEALTH-49 A and HEALTH-49 E* module A (psychological and somatoform complaints) and module E (restrictions on activity and participation) of the Hamburg Modules for the Assessment of Psychosocial Health in Clinical Practice, *FFA-14* short version of the Freiburg Mindfulness Inventory, *SCS-D* short German version of the of the self-compassion scale, *HRV-Bfb* heart rate variability-biofeedback (n = 18), *MBI* mindfulness-based intervention (n = 15), *WLC* wait-list-control-group (n = 19)

#### Differences Between the Groups at the Different Points of Measurement

There was a significant interaction effect of time by group for negative coping strategies [*F *(4, 98) = 2.83, *p* = .029, *η*² = 0.103] with post hoc tests not showing any significant differences between the groups at T0 (*p* = .639), T1 (*p* = .221) and T2 (*p* = .287). There were no other statistically significant between-group-effects for the experimental groups and the WLC at any other point of measurement for chronic stress, positive coping, HRV parameters or cortisol, see Table 2. Analysis of secondary outcome parameters showed a significant interaction between time × group for measures of mindfulness, *F *(4, 98) = 3.27, *p* = .015, *η*² = 0.118. However, post hoc tests didn’t reveal any significant differences between the groups at T0 (*p* = .379), T1 (*p* = .395) and T2 (*p* = .846) There were no statistically significant between-group-effects at any point of measurement for depressive symptoms, psychological wellbeing or self-compassion (s. Table 2).

#### Within-Group Effects

Within-group-effects were found on different parameters for all three groups (s. Table 2). Chronic stress was significantly reduced over time [*F *(2, 98) = 9.34, *p* < .001, η² = 0.16], with a significant decrease of stress between pre and post intervention for HRV-Bfb (*p* = .01), MBI (*p* = .035) and the WLC (*p* = .04). The HRV parameter SDNN showed for the short-term measurement a significant change over time [*F *(1.55, 75.98) = 5.05, *p* = .015, η² = 0.09]. Post hoc tests confirmed a significant increase in SDNN between T0 and T2 for HRV-Bfb (*p* = .01) and the WLC (*p* = .046). Cortisol changed significantly over time [*F *(2, 98) = 12.78, *p* = < 0.001, η² = 0.21] with post hoc tests confirming a decrease of cortisol for all three groups between pre and post intervention (HRV-Bfb: *p* = .016; MBI: *p* = .004; WLC: *p* = .034). We did not find any significant changes over time for the other measures of HRV or the positive and negative coping.

For secondary outcome parameters, psychological and somatoform complaints showed a main effect for *time *[*F *(2, 98) = 8.42, *p* = < 0.001, η² = 0.15], post hoc tests confirmed a significant reduction within the WLC between pre and follow-up (*p* = .015). Mindfulness scores [*F *(2, 98) = 8, *p* = .001, η² = 0.14] as well as self-compassion [*F *(1.77, 86.9) = 6.05, *p* = .005, η² = 0.11] significantly changed over time. Post hoc tests confirmed a significant increase in both parameters only within the MBI group between pre and post intervention (mindfulness: *p* = .015 and self-compassion: p = .031) and between pre and follow-up intervention (mindfulness: p = .001 and self-compassion: p = .002). Depressive symptoms and restrictions on activity and participation did not improve significantly in any of the groups.

#### Post Hoc Analysis

Because participants reported a wide range of perceived stress at baseline (range = *M* = 2–34), we suspected that change in outcome parameters for participants in the IGs would be more likely to be seen for the more highly stressed participants. We computed change scores for each of the primary and secondary outcome parameters by subtracting each participant’s pre-treatment score from their post-treatment score and pre-treatment score from their follow-up-treatment score. We then tested this post hoc hypothesis using the change scores. Stress level in the pre-test was significantly correlated with reduction of perceived stress after the intervention and significantly correlated with reduction in perceived stress and increase in activation and participation at follow-up (s. Table 4). Secondly, as there was also a relatively wide variability in daily practice time between the training and the post measurement for the intervention groups (range = 5–30 min), we speculated that greater practice time would be correlated with greater reduction of stress and related symptoms. Correlations again were computed the same way between the outcome parameters and frequency of practice between pre-treatment and post-treatment and pre-treatment and follow-up. There were no significant correlations (all *p*s > 0.05).

**Table 4:** Correlations between baseline stress and change scores of stress and related parameters

Measure	Stress level at baseline (N = 33)	
	T0–1	T0–2
TICS-SSCS	− 0.469**	− 0.482**
SVF 120		
POS	0.118	0.186
NEG	− 0.119	− 0.125
HRV		
RMSSD		
RSA	− 0.052	0.13
Short-term	0.226	0.193
SDNN		
RSA	0.024	0.018
Short-term	0.317	0.298
Cortisol	− 0.19	− 0.073
BDI-II	− 0.324	− 0.212
HEALTH-49 A	− 0.14	− 0.176
HEALTH-49 E	− 0.281	− 0.382*
FFA-14	0.369	0.116
SCS-D	0.108	0.213

*TICS-SSCS* scale *chronic stress* of the Trier Inventory for Chronic Stress, *SVF 120 POS* scale *positive coping strategies* of the stress-coping questionnaire, *SVF 120 NEG* scale *negative coping strategies* of the stress-coping questionnaire, *HRV* heart rate variability, *RSA* respiratory sinus arrhythmia, *RMSSD* root mean square of the squared differences of the RR intervals of successive heartbeats, *SDNN* standard deviation of the RR intervals, *BDI-II* Beck-depression inventory, *HEALTH-49 A and HEALTH-49 E* module A (psychological and somatoform complaints) and module E (restrictions on activity and participation) of the Hamburg Modules for the Assessment of Psychosocial Health in Clinical Practice, *FFA-14* short version of the Freiburg Mindfulness Inventory, *SCS-D* short German version of the of the self-compassion scale

*p < .05, **p < .01

## Discussion

This study compares two established methods for stress reduction (HRV-Bfb and MBI) on an organizational level to a waiting-list control group.

In line with Van der Zwan et al. (2015) and Ratanasiripong et al. (2015) we did not find any statistically significant differences between HRV-Bfb and MBI in terms of stress reduction. In within-group comparisons we detected small to medium effect sizes for all primary outcomes (perceived stress, coping and HRV parameters), except for the large effect sizes for change in cortisol for the intervention groups at T1 after the intervention. Effect sizes for psychological measures of stress are in line with effect sizes reported by other studies (Van der Zwan et al. 2015; Keeney 2008; Roeser et al. 2013). However, Goessl et al. (2017) report in their review very heterogeneous effect sizes for perceived stress after an HRV-Bfb intervention, with some studies having medium to large effects. As there are very view studies examining effects of both HRV-Bfb and MBIs on physiological parameters, it is difficult to compare our results. Rijken et al. (2016) also reported small effect sizes for HRV parameters RMSSD and SDNN after a treatment-as-usual combined with HRV-Bfb for athletes. Grossman et al. (2004) report medium effect sizes for psychological and physiological measures following an MBI. In within-group comparisons on secondary outcome parameters related to stress, we found a medium effect for the reduction of depressive symptoms in HRV-Bfb at T1, whereas at T2 there was no effect. MBI showed a large effect for a reduction in depressive symptoms at T1 and T2. Small (HRV-Bfb) to medium (MBI) effect sizes were found in the increase of psychological wellbeing. These results show for depressive symptoms slightly bigger effects than those of other studies with healthy participants, but are comparable for psychological wellbeing (Van der Zwan et al. 2015). An increase in mindfulness and self-compassion had a medium to large effect size for MBI, which is in line with other studies (e.g. Schroeder et al. 2016) reporting medium to large effect sizes. Our results show that MBIs may have a slightly stronger effect on stress reduction compared to HRV-Bfb, as suggested by effect sizes. However, these results need to be interpreted with caution due to the relatively small sample size. Mindfulness specific elements that favor a general change in attitude towards life might be an explanation for this.

Although trends in our results favored the IGs on stress reduction and are in line with findings of other mind–body workplace stress reduction interventions, there is a lack of statistically significant differences between the intervention groups and the WLC in this study. As indicated by recent reviews for HRV-Bfb and MBI, there is a lack of RCTs for both interventions (Goessl et al. 2017; Jamieson and Tuckey 2016). Especially for HRV-Bfb, few studies compare HRV-Bfb to a control group. However, these studies are more likely to find positive effects of HRV-Bfb for participants with clinically significant symptoms than for healthy controls (Henriques et al. 2011; Whited et al. 2014; Siepmann et al. 2008). The results of the post-hoc analysis indicate that our study is consistent with these findings—more highly stressed participants exhibited greater benefit. These findings raise the question, why were individuals in the two treatment conditions at both times (post, follow-up) no more likely than individuals in the wait-list control condition to report reduced stress? We suspected different factors to be possible explanations for the lack of intervention effects.

First, we suspected that the failure of the interventions to reduce stress to a greater extent was related to the fact that many individuals included in the study weren’t highly stressed before the start of the study. Although allowing all employees to potentially participate in the study was in our case the only feasible way to implement the study, we did check baseline levels of stress in order to include participants with at least average levels of stress. However, average stress levels may not be high enough to show intervention effects (De Witte et al. 2019). Post hoc analyses supported this speculation as we found significant correlations between higher levels of perceived stress before the study and decreases in primary and secondary outcome parameters of stress and related symptoms (chronic stress and participation and activation). Eisen et al. (2008) attributed their intervention’s nonimpact on global indices of stress to insufficient practice time and lack of integration of the techniques into the daily lives of employees. Mean daily practice time for both intervention groups in the present study [HRV-Bfb: *M* = 19.74 (*SD* = 7.01), MBI: *M* = 16.11 (*SD* = 6.97), in min] is in line with other studies finding effects of stress reduction interventions on parameters of stress (e.g. Bruin et al. 2016). However, the wide variability in practice time between participants in our study could have influenced the results. We tested post hoc the speculation that more frequent practice of the respective method would be related to more stress reduction, but our analyses did not support it. Future research may focus on differentiating effects of stress reduction techniques on participants with high and low stress levels and on other possible moderators. For this purpose, more subgroup analyses should be conducted in larger scale samples (Biron and Karanika-Murray 2014).

Second, participants practiced their skills over a 6-week period. One could argue that this isn’t enough time to bring about significant psychophysiological changes. However, there are studies that reported changes in HRV even after a short training period. Prinsloo et al. (2013) as well as Sherlin et al. (2009) found short-term carry-over effects after a single session of HRV-Bfb. However, these studies measured HRV during or shortly after the intervention, with no longer term follow up. It is therefore unclear whether the intervention effects were maintained. The current study included a relatively short follow-up of 12 weeks so that long-term-effects couldn’t be measured which are important regarding maintenance of the training and its effects. Effects for stress reduction at follow-up are inconsistent but the HRV parameter SDNN shows stronger effects at follow-up which could be due to a more profound training effect. Future studies may differentiate between short-term and long-term effects of HRV-Bfb by measuring HRV parameters during, shortly after, and several weeks after the training. Some studies report increased HRV only during the exposure of a stressor but not at rest (e.g. Whited et al. 2014). Therefore, it is possible that benefit of HRV-Bfb is most evident during exposure to stressors. Future studies should involve measurements during stress exposure as well as at rest. However, there also are studies which reported significant improvements of HRV after training. Tang et al. (2009) found significant increases of HRV after a MBI practice of 20 min a day for 5 days. Munafò et al. (2016) trained managers 45 min weekly with HRV-Bfb and reported improvements in autonomic function after the 5-week training. Our results suggest that the sample in the current study may be too heterogenous or insufficient in size to detect consistent changes in HRV after the trainings, compared to before the intervention.

Third, interestingly, the WLC also showed some improvements in parameters of stress and related symptoms (psychological and physiological), suggesting other factors may have influenced the results. It is possible that participants of the WLC may not truly be “untreated” and show research participation effects that might have contributed to the increase in stress reduction. Participants knew they would take part in an intervention after the IGs finished theirs, which makes it possible that the results mirror an expectation of the intervention itself. The Hawthorne effect describes this phenomenon of a positive change in measurable behavior in a situation in which there was no attentional attempt to change behavior, with change being due solely to the attention given to the participants (Mayo 1949, 1933). This is in line with the findings of some other studies. Munafò et al. (2016) for example, also compared HRV-Bfb to a control group that filled out a stress diary once a week. Psychological parameters positively changed in both groups after the intervention. Another study based in corporate health management found increased wellbeing after an intervention, even in a pure control group, suggesting non-specific intervention factors as positive group experience or social contacts to be responsible (Emrich et al. 2009). Berry et al. (2016) emphasize the possible supporting effects of employees feeling acknowledgement of their needs for support. Cook et al. (2007) suggest for their similar result of perceived stress reduction and improved coping strategies in the active control group (print media on stress reduction) that most subjects came to the study wanting to improve their health. A quasi-placebo effect could thus be responsible for the improvements. Social desirability could also have been a factor contributing to the positive change in parameters of stress and related symptoms for the WLC. Since participants knew the investigators from the first point of measurement and were welcomed in a friendly atmosphere, it is possible that they reported less stress in a subconscious attempt to meet what they perceived to be the goals of the investigators or the organization. To reduce the likelihood of such factors influencing the outcome, one would have to utilize a pure control group with no post wait intervention, as well as control for socially desirable responding. However, as improvements for the WLC consistently occurred across most of the measures, including physiological parameters, other explanations are more probable. Seasonal influences could have changed stress levels as the points of measurement varied from early spring to summer. Findings on seasonal influences on parameters of stress are, however, too inconsistent to draw any final conclusions (Denissen et al. 2008; Kristal-Boneh et al. 2000; Rennie et al. 2003; Malarkey et al. 1994; Maes et al. 1997). Additional, being a randomized controlled study, possible seasonal effects should be distributed equally in the three groups and thus should not have influenced the results. We assume structural factors of the organization itself to have partly influenced all three groups. These contextual influences are challenging for stress intervention research (Biron and Karanika-Murray 2014) because they might be stronger than the intervention effect itself. Theater is a work environment that is very dependent on the theater schedule, and it is likely that stress levels of employees are influenced by the change in schedule-dependent work load. Anderson and Pulich (2001) describe work overload as one of the most important stressors at workplace and thus a challenge for workplace interventions. Work load was reportedly heavier at the time of the first and third point of measurement, a possible explanation for the decrease and increase of most parameters of stress in all three groups at post and follow-up, as can be seen in the observed means (s. Table 3). Other studies found a similar effect of organizational structural factors influencing stress levels of employees (e.g. Wolever et al. 2012). In the context of structural influences, individual vs. organization-based approaches in occupational health management should be considered. This study is an individual-level intervention as it aims to reduce stress through individual coping. The results however hint at the need to address both intervention levels (s. reviews of Tetrick and Winslow 2015; Ivandic et al. 2017). Additionally, a company with lesser variability in workload that could result in different levels of stress should be chosen for future studies. One positive conclusion that could be drawn from the benefit seemingly derived by the WLC is that an intervention itself offered in the workplace can, at least partly, have a positive outcome in stress reduction. This could encourage occupational health management to implement stress reduction interventions in the workplace more often.

Fourth, there are some limitations to be considered. As described by Tetrick and Winslow (2015) in their review on workplace stress management interventions as one of the major challenges, recruitment to the study proved harder than anticipated. Despite the overall positive reception of the invitation to participate in a stress reduction training, employees often told us that they would not be able to participate despite wishing to do so. Practical considerations, particularly time constraints, were most frequently cited as reasons for inability to participate. Theater schedule, which tends to be harsher and more constraining than in other organizations, may be responsible for this challenge. Another contributing factor could have been that in this study employees had to proportionate take vacation time to participate at the trainings during worktime. This time conflict is unfortunate, as it shows employees may be motivated to participate in a workplace stress reduction program, with practical concerns preventing them from doing so. As intervention completion was very high (HRV-Bfb = 100% and MBI = 93.3%), we can assume that the interventions were appreciated. Future studies might try to further adapt the interventions and their settings to participants’ practical needs to encourage greater participation. This illustrates once again the importance of organizational-level-based interventions alongside individual-level-based approaches. Carson et al. (1999) discuss the possible favorable effect of employees of the same work unit participating in an intervention study by encouraging and motivating each other. In our case, employees of very different work units were included. This increases generalizability to other work contexts but reduces the possible camaraderie effect. Future studies might consider including whole work units in stress reduction interventions. It could also be an improvement for further studies to include an evaluation of the participants perception of the quality of the implementation and/or participants’ readiness to change (Biron and Karanika-Murray 2014). Inability to have a double-blind control in this study could also have impacted the results. It is possible that the researchers’ knowledge of participants group assignment could have influenced their behavior. In this study, the preponderance of female participants and the highly specialized workplace context limit the generalizability of the results of this study to the general population of working employees. Finally, the relatively small sample size may be responsible for lack of significant findings in this study. Although a priori power analysis confirmed the sample size as sufficient, the study could have been underpowered, increasing the probability of type II errors and not observing existing differences between groups. Therefore, these results need an evaluation with larger scale RCTs in order to improve its power.

The current study has a practical application as it is a field study. It highlights challenges with implementing such interventions in a context of occupational health. The use of both psychological as well as psychophysiological measurements for stress reduction to compare effectiveness of HRV-Bfb and MBI to a WLC is a novelty of the study.

Overall, both interventions seem to have a positive impact on stress reduction of employees which could prevent further stress associated diseases. Both interventions are relatively easy to implement and are therefore suitable for the context occupational health. However, findings are inconsistent and need to be studied with larger scale RCTs. Participants with higher stress levels might profit more from a reduction in psychological parameters of stress and its related symptoms.
